# Provider and client perspectives on maternity care in Namibia: results from two cross-sectional studies

**DOI:** 10.1186/s12884-018-1999-3

**Published:** 2018-09-05

**Authors:** Jennifer Wesson, Ndapewa Hamunime, Claire Viadro, Martha Carlough, Puumue Katjiuanjo, Pamela McQuide, Pearl Kalimugogo

**Affiliations:** 1 IntraHealth International, 6340 Quadrangle Drive, Suite 200, Chapel Hill, NC USA; 2 Ministry of Health and Social Services, Windhoek, Private Bag 13198 Namibia; 3 Independent consultant, Chapel Hill, USA; 4 Department of Family Medicine, University of North Carolina School of Medicine, Chapel Hill, NC USA; 5 IntraHealth International, 351 Dr. Sam Nujoma Dr., Klein Windhoek, Windhoek, Namibia

**Keywords:** Childbirth, Institutional delivery, Respectful maternity care, Disrespect and abuse, Quality of care, Maternity care workers, Nurses, Workload

## Abstract

**Background:**

Disrespectful and abusive maternity care is a complex phenomenon. In Namibia, HIV and high maternal mortality ratios make it vital to understand factors affecting maternity care quality. We report on two studies commissioned by Namibia’s Ministry of Health and Social Services. A health worker study examined cultural and structural factors that influence maternity care workers’ attitudes and practices, and a maternal and neonatal mortality study explored community perceptions about maternity care.

**Methods:**

The health worker study involved medical officers, matrons, and registered or enrolled nurses working in Namibia’s 35 district and referral hospitals. The study included a survey (*N* = 281) and 19 focus group discussions. The community study conducted 12 focus groups in five southern regions with recently delivered mothers and relatives.

**Results:**

Most participants in the health worker study were experienced maternity care nurses. One-third (31%) of survey respondents reported witnessing or knowing of client mistreatment at their hospital, about half (49%) agreed that “sometimes you have to yell at a woman in labor,” and a third (30%) agreed that pinching or slapping a laboring woman can make her push harder. Nurses were much more likely to agree with these statements than medical officers. Health workers’ commitment to babies’ welfare and stressful workloads were the two primary reasons cited to justify “harsh” behaviors. Respondents who were dissatisfied with their workload were twice as likely to approve of pinching or slapping. Half of the nurses surveyed (versus 14% of medical officers) reported providing care above or beneath their scope of work. The community focus group study identified 14 negative practices affecting clients’ maternity care experiences, including both systemic and health-worker-related practices.

**Conclusions:**

Namibia’s public sector hospital maternity units confront health workers and clients with structural and cultural impediments to quality care. Negative interactions between health workers and laboring women were reported as common, despite high health worker commitment to babies’ welfare. Key recommendations include multicomponent interventions that address heavy workloads and other structural factors, educate communities and the media about maternity care and health workers’ roles, incorporate client-centered care into preservice education, and ensure ongoing health worker mentoring and supervision.

**Electronic supplementary material:**

The online version of this article (10.1186/s12884-018-1999-3) contains supplementary material, which is available to authorized users.

## Background

Over the past five years, global health experts have accelerated their efforts to build a respectful maternity care (RMC) movement to counteract disrespect and abuse by health workers during pregnancy and childbirth, particularly in low- and middle-income countries (LMICs) [1–3]. The phenomenon of disrespectful and abusive maternity care is complex, arising from systemic and policy failures and health worker behavior. Perceptions of disrespect and abuse are shaped by local norms and health worker intentions in addition to women’s experiences [1, 2]. As a response, RMC encompasses not just the absence of disrespect and abuse but also the presence of “positive and supportive staff attitudes and behaviors that increase a woman’s satisfaction with her birth experience” [2].

Numerous studies have documented disrespectful and abusive maternity care from the client’s perspective, assembling evidence that suboptimal care is widespread [4–11]. A study in one region of Nigeria found that over one-third (36%) of laboring women had experienced physical abuse at the hands of health workers [9], and an Ethiopian study showed that nearly all women in childbirth (95%) had had their rights to information, informed consent, and informed choice violated [4]. Where disrespect and abuse are prevalent, additional negative consequences can result. In the short term, these may include client dissatisfaction, ineffective communication of health promotion messages, and poor quality of care (defined across dimensions such as safety, equity, timeliness, and client-centeredness); in the longer term, women may be less likely to seek institutional care, potentially increasing the risk of adverse maternal and newborn health outcomes [6, 12–16]. A qualitative gender assessment in Ethiopia that explored gender barriers to facility-based delivery in the context of prevention of mother-to-child transmission of HIV services found that disrespect and abuse was the primary deterrent to institutional delivery cited by community members [17].

Studies focusing on clients’ experiences of institutional childbirth are clearly important, but there is a complementary need to examine disrespect and abuse from the health worker’s perspective to understand underlying factors that propel negative attitudes and practices. A systematic review of 81 peer-reviewed studies conducted in LMICs, published between 1990 and 2014 and cited in at least one of five electronic databases, examined both positive and negative attitudes and behaviors of maternal health care providers (including nurses, midwives, physicians, and other maternity care cadres), as reported by clients, health workers, or others [13]. Over two-thirds of the studies (68%) took place in Africa, with the remainder set in Asia and the Pacific, Latin America, or the Middle East [13]. Thirty-one studies (38%) elicited feedback from health workers, most of which were exploratory and relied primarily on qualitative methods [13]; only one study used survey methods exclusively [18]. The evidence furnished by the review [13] and other studies [2, 5, 7, 8, 11, 19–23] suggest that health system factors such as understaffing, overwork, inadequate pay, and suboptimal training and supervision—factors “embedded” in the broader sociopolitical context and power dynamics [1]—play a substantial role in shaping disrespectful and abusive attitudes and practices among maternity care workers. Where working conditions are subpar, health workers may experience stress, exhaustion, and frustration, which in turn can lead to “compassion fatigue” toward laboring women [11, 13, 24]. Other structural factors may also play a role. One study found an association between verbal/physical abuse and night shift deliveries due to lower nighttime staffing and less “patient, companion, co-worker and management pressure to adhere to…norms” [19]. A rigorous consideration of maternity care-related disrespect and abuse clearly should identify health worker’s attitudes and behaviors *and* systemic deficiencies [1].

In Namibia, a lively media market and freedom of the press are strongly established (if imperfect) features of civil society [25]. Numerous media reports have criticized poor quality health care and client mistreatment by health workers, making health workers a target of popular anger [26, 27]. In 2013, a Presidential Commission of Inquiry report on the health sector agreed that “unacceptable” conduct and “don’t care” attitudes were widespread among health workers but suggested that poor behavior might result, in part, from overwork and burnout [28]. In the context of the country’s ongoing HIV epidemic, along with a worrying maternal mortality ratio (265 deaths per 100,000 live births) and neonatal mortality rate (15.9 deaths per 1000 live births) despite a high level (87%) of institutional births [29], it is vital to understand factors that influence or diminish the quality of maternity and neonatal care.

In late 2013, Namibia’s Ministry of Health and Social Services (MoHSS) commissioned a study to identify cultural and structural factors that affect health workers’ attitudes and practices in delivering maternal and neonatal health care. The study (carried out by IntraHealth International, the Tulipohamba Training and Assessment Institute, and the University of Namibia) followed on the heels of another MoHSS study (conducted by IntraHealth and the University of Namibia in early 2013) that focused on facility-based maternal and neonatal deaths [30]. The mortality study included an ancillary qualitative component that explored community perceptions concerning delayed and poor-quality care at public institutions. This article shares key findings from the health worker study and augments those findings with selected qualitative results from the maternal and neonatal mortality study.

## Methods

### Health worker study

#### Study design

The study combined quantitative and qualitative techniques to profile knowledge, attitudes, and practices of health workers in district and referral hospitals with regard to maternity care and outcomes. From November 2013–January 2014, a survey was administered in the reference hospital in Windhoek and all 34 district hospitals in Namibia. Over the same time period, 11 of the 35 facilities participated in focus group discussions and a handful of in-depth interviews. In the 24 survey-only facilities, fieldwork generally was completed in one day per facility. In the 11 facilities where both survey and focus group data were gathered, fieldwork occurred over a two-day period per facility. The study included four categories of health workers (medical officers, matrons, registered nurses, and enrolled nurses) who had managed maternity wards or attended mothers for delivery and postnatal/neonatal care in the previous three months. In Namibia, enrolled nurses complete a two-year course of study, and registered nurses generally complete a four-year university nursing diploma or degree [31].

#### Survey

The questionnaires were administered by trained research assistants or, in some instances, self-administered in a private space in the health facility. A 172-item survey instrument was developed specifically for this study, blending original measures with items from existing scales [32]. The questionnaire covered health workers’ background characteristics, job satisfaction, barriers and facilitators to providing maternity care, regulatory frameworks, and clinical knowledge, primarily using Likert scales (ranging from “strongly agree” to “strongly disagree”) as well as some “true/false” knowledge questions (Additional file 1). Respondents to the survey constituted a convenience sample of those personnel working on the day and night shifts in the maternity wards on the day of data collection. Although the survey instrument was not validated, due to resource and time constraints, the research team drew on its familiarity with the relevant literature and the Namibian context to develop items capturing attitudes about disrespect and abuse. To minimize the potential for socially desirable responses, those items asked respondents to rate colleagues’ rather than their own attitudes and behavior. The survey also included 11 items exploring discrimination. Most of the job satisfaction items came from validated scales developed to measure job satisfaction among health professionals in sub-Saharan Africa [32].

#### Focus groups and in-depth interviews

The qualitative portion of the study aimed for two focus groups per facility. Recognizing status differences between cadres, every effort was made to conduct separate focus groups with registered versus enrolled nurses. To streamline data collection, the research team contacted maternity managers in advance of the qualitative fieldwork, and they invited separate groups of enrolled and registered nurses to participate in focus group discussions. Most maternity managers (i.e., matrons) and medical officers were interviewed individually. The data collectors conducted individual interviews with those managers and officers who were available during the two-day visit. Trained research assistants (distinct from those administering the survey) conducted the discussions and interviews in boardrooms or private offices, drawing on a two-part discussion guide focusing on (1) job satisfaction and (2) experiences providing maternity and neonatal care. For both topics, focus group moderators first asked participants to “free list” all ideas and then asked them to group ideas into summary topics for discussion by topic. To elicit ideas about job satisfaction, moderators encouraged participants to think about “everything that would make nurses feel more motivated, more productive, and more satisfied with their work.” Probes included, “Why do you think this would increase nurses’ job satisfaction?” and “How do you think this item could be integrated into the current health care system?” The second broad topic asked participants to view themselves as “nurse ambassadors” and talk about “some of the behaviors or attitudes that you have seen when you have worked in maternity wards that might be considered by an outsider to be ‘bad.’” Probes included questions such as, “Have you ever seen a health worker yell at (or ignore) a woman in labor?” and also, “As a nurse ambassador, could you explain [why this happens] from the point of view of the nurse?” All focus groups and interviews were captured through audio-recordings and written notes.

### Maternal and neonatal mortality study

#### Study design

Commissioned and approved by the MoHSS, a multisource survey of facility-based maternal and neonatal mortality was undertaken in the five southern regions of Namibia from March–May 2013, retrospectively covering the period from January 2010–June 2012 [30]. The study’s main objective was to identify the prevalence of and contributors to facility-based maternal and neonatal deaths. Methods included verbal autopsies [33] with the closest relative or contact of maternal deaths reported at the selected health facilities. In addition, focus group discussions (at least two per region) explored community perceptions of factors contributing to reduced quality and delayed provision of maternal and neonatal care. This paper reports on the focus group results.

#### Focus groups

Recruitment of focus group participants used snowball (convenience) sampling, starting with selected verbal autopsy respondents (relatives or contacts of identified maternal deaths). Respondents who completed a verbal autopsy were asked to participate in the focus groups as well as to identify additional individuals meeting two criteria: (1) delivery at the designated health facility within the past 12 months; (2) residence less/more than 50 km (km) from the facility. Using this process, each focus group included one relative or contact of a deceased woman plus recently delivered mothers (living < 50 km and > 50 km from the facility). A small number of groups also included husbands of women who had delivered in the past 12 months. Focus group data were recorded and transcribed in the original language and entered into Microsoft Word.

### Analysis

The health worker survey data were analyzed using descriptive statistics in SPSS 21.0. Cross-tabulations by cadre and age group were conducted for key variables. The focus group and interview data from the health worker study were summarized in field notes by listening to the audio recordings, referring to notes, and requesting clarification from the field research team where needed. The first author (JW) and an IntraHealth colleague used a conventional content analysis approach [34] to code the data according to the key themes that emerged in the free listing and topic grouping exercise, and explored additional themes through a process of memo-writing and discussion. After coding the data independently, the two exchanged the coded data to ensure reliability, discussing and resolving areas of disagreement where needed. For the focus group data from the maternal and neonatal mortality study, the in-country field team used a more directed content analysis approach [34] to select short passages illustrating the key themes of interest (i.e., factors contributing to reduced quality and delayed provision of maternal and neonatal care) and translated the passages into English. The same two individuals (JW and IntraHealth colleague) then reviewed the passages and placed them into defined coding categories. Where there were discrepancies, the two discussed and reached agreement on which codes to use for particular segments.

## Results

### Study participants

A total of 281 health workers spanning all 35 district and referral hospitals in Namibia completed the health worker survey. In the 11 hospitals participating in the qualitative component, the research team conducted six individual interviews and 19 focus group discussions with 96 health workers (range = 3–7 participants), separated to the extent possible by cadre. Of the survey respondents 256/281 (91.1%) were female as well as 93/102 (91.1%) of the qualitative participants. Of the survey respondents 267/281 (95.0%) were enrolled or registered nurses as were 82/102 (80.4%) of the focus group/interview participants (Table 1). Survey respondents had an average of ten years of professional experience, most of it in maternity care. Of the survey respondents 47/280 (16.8%) reported ever having worked in the private sector, but 215/273 (78.8%) stated that they would not take a job in the private sector, even if it offered the same salary.

**Table 1:** Health worker study: participant characteristics

Characteristics	Survey (*n* = 281)	Focus Groups/Interviews (*n* = 102)
Sex		
Female	256 (91.1%)	93 (91.2%)
Male	25 (8.9%)	9 (8.8%)
Mean age	37.4 years	39.5 years
Cadre		
Medical officers	14 (4.9%)	12 (11.8%)
Matrons	–	8 (7.8%)
Registered nurses	156 (55.5%)	53 (52.0%)
Enrolled nurses	111 (39.5%)	29 (28.4%)
≥ 5 years’ experience^a^		
All health care	159 (61.9%)	–
Maternity care	135 (52.3%)	–
Mean years’ experience		
Medical officers	11 years	13 years
Matrons	–	23 years
Registered nurses	11 years	14 years
Enrolled nurses	7 years	13 years

^a^Due to missing data, the denominator is *n* = 257 (all health care) and *n* = 258 (maternity care), rather than 281

The maternal and neonatal mortality study conducted 12 community focus groups with 109 women (86.2%) and 18 men (13.8%). Eight groups were made up of women only, and four groups included both women and men. In general, men proved difficult to recruit, offering excuses such as being unable to “comment on any pregnancy-related instances” or unwilling to “discuss my involvement with the mother of my child.” Some female participants also were unwilling to have their male partners participate. Focus groups ranged in size from 4 to 19 participants (average size = 11 participants).

### Vocation

As a starting point for understanding health workers’ point of view, a survey item included at the behest of the MoHSS asked respondents what motivated them to become a health worker. Seven in ten survey respondents (184/325; 56.6%) reported choosing their career in response to a perceived “calling,” and another 56/325 (17.2%) reported having been motivated by “a personal experience with the health care system.” (Respondents could choose more than one response.) Relatively few respondents cited reasons such as availability of jobs (32/325; 9.8%) or salary and benefits (5/325;1.5%). Over three-fourths (211/273; 77.3%) indicated that they would choose the same career if they “had to decide over again”; older health workers (≥ age 40) (*p* < 0.01), and those who chose their career because of a calling or personal experience (*p* < 0.001) were more likely to *strongly* agree that they would choose the same career again.

### Attitudes and behaviors related to maternity care

Both the quantitative and qualitative components of the health worker study asked health workers to reflect on colleagues’ provision of maternal and neonatal health care. Some participants volunteered first-person accounts in the focus group discussions. When asked whether they thought most colleagues were respectful of maternity ward patients, the majority (231/278; 83.1%) of survey respondents agreed (Table 2). However, one in three respondents (86/276; 31.2%) reported witnessing or knowing of client mistreatment at their institution.

**Table 2:** Selected health worker attitudes about maternity care, all cadres (survey results)

Survey item	Number and percent agreeing
I feel most of my colleagues are respectful of patients when providing maternal and neonatal health care (*n* = 278).	231 (83.1%)
I have seen colleagues or know of incidences when patients were mistreated at my institution (*n* = 276).	86 (31.2%)
Patients complain too much, they don’t understand how hard we are working (*n* = 277).	220 (79.4%)

Among survey respondents, 137/277 (49.5%) agreed that “sometimes you have to yell at a woman in labor because she is not pushing hard enough,” and 82/272 (30.1%) agreed that “pinching or slapping a woman in labor can succeed in getting her to push harder” (Table 3). Registered and enrolled nurses were much more likely to agree with the statement about yelling than medical officers (51–52% versus 15%; *p* = .012), and only nurses (but no medical officers) agreed with the statement about pinching or slapping (Table 3).

**Table 3:** Selected health worker attitudes about maternity care, by cadre (survey results)

Survey item	Percent agreement (strongly agree or agree)			
	MO	RN	EN	All
Sometimes you have to yell at a woman in labor because she is not pushing hard enough (*n* = 277).	15.3%	51.8%	51.1%	137 (49.4%)
Sometimes pinching or slapping a woman in labor can succeed in getting her to push harder (*n* = 272).	–	32.9%	30.5%	82 (30.1%)

*MO* Medical officer; *RN* Registered nurse; *EN* Enrolled nurse

The perceived necessity of yelling at laboring women to encourage pushing was mentioned in every focus group discussion and most interviews. Nurses in the focus groups also reported other disrespectful behaviors (such as hostile body language, intimidation, and “beating”), but often linked these to patient behaviors such as being “uncooperative” or unwilling to “open their legs.” First-time mothers were singled out as being the least cooperative due to their inexperience. One participant remarked:

There are patients who do not want to cooperate, and it results in nurses beating the mother who does not want to open her legs to save the baby*.*

A primary reason offered for disrespectful behavior was the need to take forceful action “for the sake of the baby.” Across focus groups, both cadres of nurses discussed—with visible emotional intensity—the topic of babies’ lives being at stake, suggesting that worries about adverse neonatal outcomes create a need to use any means necessary to avert such outcomes. One nurse passionately and frankly stated, “Sometimes I end up beating the mother because I cannot watch her killing the baby!” Another nurse described the gradual professional socialization that shapes this attitude:

…I saw a nurse yelling at a patient…when I was a student and I thought that [was] rude then. But when I became a nurse, I understood why we have to yell sometimes because it is about saving a life. Because if something goes wrong, you are blamed*.*

Excessive workloads and stressful working conditions were the second major reason cited by focus group and interview participants to explain short-tempered and “harsh” behaviors toward women in labor. As one nurse commented, “If I am tired and hungry, you will not see a smile on my face.” Most (220/277; 79.4%) survey respondents agreed that clients “don’t understand how hard [health workers] are working” and “complain too much” (Table 2). A focus group participant commented, “We put so much effort in our work even though there is a shortage of staff, but we are not recognized.” Focus group and interview participants also bitterly described charged situations where clients or relatives are convinced that health workers are doing the wrong thing despite the health workers’ good intentions.

Roughly half or more of the survey respondents agreed that it was easier to provide care to women with more education (118/279; 42.3%) or from their same ethnic (129/277; 46.6%) or language (180/277; 65%) group, but few reported that 28/276 (10.1%) pregnant women under age 20 or) or 61/280 (21.8%) women of low social status were routinely treated in a discriminatory manner compared to other women (Table 4). These types of discrimination were not major topics of discussion in the health worker focus groups.

**Table 4:** Selected health worker attitudes about clients, all cadres (survey results)

Survey item	Number and percent agreeing
When a woman speaks my language, it is easier for me to assist her during labor and delivery (*n* = 277).	180 (65.0%)
It is easier for me to assist a woman from my ethnic group (n = 277).	129 (46.2%)
It is easier assisting educated women when they come for maternal and neonatal care than women who are not educated (*n* = 279).	118 (42.3%)
Some providers at this facility treat women of low social status more poorly than other women of higher status (*n* = 280).	61 (21.8%)
Pregnant women less than 20 years old are sometimes treated negatively compared to the treatment of older women in my health facility (*n* = 276).	28 (10.1%)

### Health system deficiencies

Health workers’ dissatisfaction with heavy workloads emerged as a key theme in the survey, focus groups, and in-depth interviews. Only 69/279 (24.7%) of those surveyed agreed with the statement, “I am satisfied with the workload that I have” (Table 5). Of the survey respondents, 103/279 (37.0%) were, in fact, strongly *dissatisfied* with their workload. Few respondents agreed that “There is an adequate number of staff at this hospital to provide quality care” during either the day shift (53/279;18.9%) or night shift (31/279; 11.1%). These responses were echoed across the focus groups and interviews, where participants expressed frustration with the perceived high client-to-staff ratios prevailing in hospital maternity wards, and one group loudly chanted, “More staff, more staff!” Some health workers commented that many nurses do not want to work in maternity wards because the workload there is higher than in other hospital units.

**Table 5:** Selected health worker attitudes about workload, all cadres (survey results)

Survey item	Number and percent agreeing
I am satisfied with the workload that I have (*n* = 279).	69 (24.7%)
There is an adequate number of staff at this hospital to provide quality care:	
-Day shift (*n* = 279)	53 (19.0%)
-Night shift (*n* = 279)	31 (11.1%)
I am satisfied with the amount of my salary as compared to my workload (*n* = 281).	57 (20.3%)
Sometimes I have served so many pregnant women that I am too tired to give the next woman good service (*n* = 275).	118 (43.0%)

Survey respondents who were dissatisfied with their workload were twice as likely to agree that sometimes pinching or slapping a woman can succeed in getting her to push harder (30% dissatisfied; 15% satisfied; *p* < .01). A focus group participant commented, “Due to a lot of patients, you are under pressure and you end up shouting.” Another health worker also emphasized the impact of stressful workloads:

*Due to pressure and too many patients, sometimes nurses have no choice but to ignore patients…and attend to others in the waiting room, and also, depending on the situations, the nurses may respond to patients without thinking.*

When comparing workload against remuneration, only 57/281 (20.3%) of survey respondents reported being “satisfied with the amount of my salary as compared to my workload” (Table 5). However, survey items that asked about salary uncoupled from workload elicited less dissatisfaction. Salary was not a top complaint in the focus group discussions, but some participants reported being underpaid for the kind and amount of work done. As one participant remarked, “If we [were] paid according to the patients that we care for, you will see us being friendly.”

Most survey respondents reported being satisfied with the quality of their work (246/279; 88.1%) and the outcomes of deliveries in their units (251/278; 90.2%). At the same time, however, study participants consistently linked understaffing and long hours to lower quality care. More than two-fifths of surveyed health workers (118/275; 42.9% or), for example, reported sometimes being “too tired to give the next woman good service” (Table 5). As another consequence of staffing shortages, focus group participants frequently reported providing care outside of their scope of work. These tasks might include duties ordinarily carried out by less qualified staff as well as duties above enrolled and registered nurses’ professional level. In a focus group, one nurse stated, “We are clerks, we are porters…, you have to be a cleaner as well. We are doing multi-tasks.” Of greater concern than performing menial tasks, nurses described “covering” for busy or absent physicians: “We end up taking risks to help out because doctors are very busy.” Enrolled nurses with two years of training also reported carrying out duties that should be performed by registered nurses with four years of training: “Once at work, we do everything, even complicated cases in the scope of the registered nurse. There is no difference between a registered or enrolled nurse.” Overall, only half of the survey respondents reported being satisfied with the fit between their job description and “what I actually do.”

The survey and focus group discussions also covered other elements of the work environment. Regarding supervision, 70/277 (25.2%) of survey respondents did not perceive their managers as supportive and approachable or adequately present (78/275, 28.4%). Many focus group participants agreed that better-quality supervision is needed. Noting that the media’s focus on negative maternal care outcomes damages health workers’ reputation, focus group participants articulated a desire for more recognition and positive feedback from superiors and expressed disappointment with the lack of MoHSS and community support in the face of media criticism. As one health worker commented, “We are human beings, mistakes will always happen, but even a ‘Thank you’ for what we are doing is not there.”

### Client and community perceptions

Results from the focus groups carried out for the maternal and neonatal mortality study provide a counterpoint to the health worker results, illustrating community frustration about health workers’ attitudes and behaviors and quality of care. Across groups, the discussions elucidated 14 distinct types of negative practices affecting clients’ experiences of maternity care (Table 6). Recognizing that it is not always straightforward to classify specific practices as due to health worker behavior or systemic failures [2], Table 6 categorizes some practices as primarily systemic, some as both systemic and health-worker-related, and some as primarily related to health workers’ attitudes and skills.

**Table 6:** Client perceptions of negative practices in maternity and neonatal care (focus group results from maternal and neonatal mortality study)

Negative practice	Level	
	Systemic	Health worker
Facility understaffing	X	
Ambulance delays	X	
Lack of privacy/confidentiality	X	X
Delayed intake	X	X
Not adequately responsive to labor progress	X	X
Abandonment/unattended birth^a^	X	X
Clients required to do housekeeping	X	X
Providers rude or disrespectful		X
Deliberately delayed care		X
Language/tribal barriers		X
Discrimination (young people)		X
Physical abuse		X
Inappropriate care/discharge		X
Mismanagement of pain		X

^a^*Hastings* [2] *points out, for example, that staff abandonment of a woman during childbirth “could be due to health providers’ disregard of her needs, or it could be a result of poor client-to-provider ratio”*

Systemic factors topped the list of negative practices in terms of the number of focus groups in which they were mentioned. Clients generally shared health workers’ opinions that facilities are grossly understaffed—both overall and by medical officers, in particular. Participants consistently reported only one or two health workers being on duty at a given time in maternity units. Although staff shortages often resulted in delayed attention, some participants expressed sympathy for the tired and hungry health workers who work “day and night.”

Some of the health-worker-level barriers that were cited are likely to be partially or even strongly related to chronic understaffing. These practices include delayed intake, unattended births, and requests for clients to do their own housekeeping (Table 6). One mother’s description of her birth experience illustrates these themes:

*When the labor pains started, I came to the hospital and there was only one nurse on duty. There was one lady in labor already. I went to the delivery room as I felt the baby was coming. The nurse told me to wait as he was busy with the other girl. I gave birth on my own. He only came back after delivery and assisted me with cleaning the room. I did not have a choice but to clean my baby myself. …The staff complement is not enough for the maternity ward.*

Two of the health-worker-level practices—inappropriate care/discharge and mismanagement of pain—reflect possibly inadequate clinical skills but were not discussed at great length. The remaining five health-worker-level practices pertain more directly to health workers’ attitudes and behaviors, with rude and disrespectful behavior toward mothers generally (and/or toward young mothers in particular) attracting mention in all 12 focus groups. Community members described how “nurses talk to patients any way they like,” “are always scolding,” and “shout [at] and insult” laboring women. One woman reported that “the nurse scolded at me in public that it was my stupidity that I fell pregnant.” In five focus groups, participants also described physical abuse. For example, a participant reported having observed ill-treatment of a 17-year-old mother, using nearly identical language about “beating” and “opening her legs” as used in the health workers’ focus groups:

*They even beat her so she could push and do as they said. It was confusing for her. She gave birth, but the baby died the next day. The nurses claimed that the girl did not want to open her legs….*

Although it is not possible to make causal inferences from focus group data, the findings suggest that women’s concerns about health worker staffing and competence may contribute to delayed care-seeking or avoidance of facilities. One participant reported, “I…told my uncle that I would only go to the hospital once my water broke. I did not want to stay long in the hospital because the nurses were not very patient with patients.” Another stated that some women “choose to give birth at home because they are not treated well at the hospital.”

## Discussion

Both studies indicate that in Namibia’s public sector hospitals, maternity units are high-stress environments that more often than not burden health workers with heavy workloads and confront them and clients with structural impediments to quality care. Health workers, clients, and relatives reported that negative interactions with laboring women (such as ignoring requests for care or being rude, disrespectful, or physically abusive) were common, despite a high level of passionately expressed health workers’ commitment to babies and their welfare. Indeed, health workers who felt their workload was too high were more likely to agree that pinching or slapping a woman in labor might encourage her to push harder. When underpaid, overworked, and under-supported maternity care workers perform in unsafe, poorly equipped, or crowded labor wards, it is not altogether surprising that it may “beget negative experiences” [3], including demoralization of health workers and dehumanization of clients [7].

Both understaffing and unsanctioned task sharing are acknowledged problems that Namibia’s MoHSS is working hard to address [35]. Among survey respondents, half of the nurses (but few medical officers) reported providing care both below and above their scope of work. Enrolled nurses explicitly described doing the work of more highly trained registered nurses, and others used words such as “multi-tasking,” “covering,” and “taking risks” to describe the lack of coherence between job descriptions and actual duties. As a chronic problem, this mismatch likely contributes to maternity care workers’ frustration with working conditions and salaries.

Although structural constraints do not excuse disrespectful and abusive behavior, which is a violation of women’s human rights, they do suggest that interventions to promote respectful maternity care require a “societal contribution, at both policy and community levels” [19]. Adopting a broader perspective implies “calling out” disrespect and abuse “for what it is,” namely, a “symptom of fractured health systems and locally expressed power dynamics that conspire against both patients and providers” [36]. Power dynamics did not emerge as an explicit theme in the Namibian studies, but studies elsewhere have identified “abusive hierarchical management structures” [37], workplace humiliation [21], and “a sense of professional inadequacy and inferiority” [23] as constraints faced by nurses/midwives and female health workers, in particular. A study in Egypt found that because of power differentials between physicians and nurses, physicians were significantly less receptive to the potential for collaborative professional relationships than were nurses [38]. The finding that nurses were far more likely than medical officers to agree with statements about the necessity of yelling at, pinching, or slapping women in labor suggests that professional hierarchies and stereotyped roles may play a part in shaping Namibian nurses’ attitudes and in-the-trenches behavior. However, one cannot ignore the fact that nurses also spend far more time with clients during labor than do medical officers, increasing the potential for short-tempered behaviors to be triggered. As one response, nurses and midwives may need to embrace the role of change agent rather than seeing themselves as “victims of external and internal causal relationships over which they have no influence” [23]. A supportive environment is also crucial, as some obstacles can only be addressed at higher levels [21, 23, 37].

The majority of surveyed health workers reported entering health professions in response to a vocational calling (70%) and hypothetically agreed that they would choose the same career again (77%). These results compare favorably with a three-country qualitative study in Burkina Faso, Ghana, and Tanzania, in which health workers reported a strong sense of vocation and duty and shared the view that health work is an “honorable” profession [10]. However, in contrast to Ghanaian respondents, who reported “considerable admiration and status…attached to being a health professional” [10], many health workers in Namibia expressed anger and disappointment about the recurrently negative focus of the media, the public, and politicians on “sensational critical incidents” of medical neglect [39]. The emergence of civil society as a third regulatory power (in addition to professional self-regulation and government regulation) that “shapes the way health care providers interact with…clients and communities” [39], though neither unique to Namibia nor something to be discouraged, can pose challenges to health workers when discontent and mistrust are the principal outcomes. While civil society’s watchdog function is important [39], health workers in Namibia clearly articulated a desire for a voice in the media to express their realities and for meaningful recognition of their frontline contributions from community members, supervisors, and higher-level officials. Community appreciation can significantly enhance job satisfaction [10], and studies indicate that it is possible for health workers to take pride in helping their communities even under the most difficult circumstances [11].

There is no doubt that cultural factors influence health worker-client interactions on maternity wards. For example, studies indicate that health workers and clients may have differing perceptions about the expression of pain and pain management [40]. A particularly significant cultural barrier to address disrespect and abuse may be its normalization by clients, health workers, or both [1, 4, 7, 41, 42]. From the client side, a study in Ethiopia found that 79% of women objectively had faced at least one form of disrespect or abuse during childbirth, but only 16% subjectively identified any disrespect or abuse [4]. From health workers’ perspective, the Namibian survey and focus groups furnish evidence of normalization via health workers’ comments about saving babies’ lives at all costs. Under potentially life-threatening circumstances, health workers may frame behaviors such as beating or slapping as necessary forms of “discipline” rather than as “abuse” and may be less likely to identify such behaviors as unacceptable [2, 6, 42, 43]. Unfortunately, allowing an “underpinning ideology of clients inferiority” and accompanying coercive and punitive actions [41] to remain unchallenged has the potential to perpetuate a negative cycle in which abuse becomes expected, the expectation of abuse engenders negative attitudes about health facilities, and the negative attitudes ultimately discourage women from seeking institutional care during childbirth [8]. Anecdotal accounts in many contexts report that the care received by clients of private health care facilities is more respectful than that of public-sector clients. Further inquiry could examine if this is objectively true and assess what it is about the private sector setting that facilitates more respectful care.

Vogel and coauthors note that efforts to prevent mistreatment are not necessarily the same as efforts to promote respectful care [3]. For example, a study in India that compared client and health workers’ perspectives found that while interpersonal behaviors (e.g., respect, dignity, privacy, sharing of information) were central concerns for clients, health workers did not view them as “key aspects of care which needed immediate attention” [20]. Similarly, few health workers in the Namibian study spontaneously brought up interpersonal elements of RMC such as non-judgmental and culturally sensitive attitudes, the ability to listen and provide reassurance, or an approachable interaction style [15]. One group of intervention researchers has suggested that the challenges of eliminating disrespectful and abusive care and actively fostering RMC are best tackled through a collaborative rather than confrontational approach [44]. These authors observe that while there is a clear power differential between health workers and clients, “it is also true that the status quo is often maintained, not necessarily by a desire on the part of health workers to hold on to privileges and exploit users, but often just by poor information flows and lack of understanding of shared needs and priorities” [44]. Identifying areas where health workers and clients can “collectively solve problems that plague local health services” offers a fruitful avenue for intervention [44]. Some clients in the Namibian focus groups were able to transcend their own experience and acknowledge that maternity care workers were overworked—echoing a Tanzanian study that showed that female clients empathized with overburdened providers [8]. This finding lends support to the potential for collaborative and positive interventions.

### Limitations

The lack of comments about RMC or other positive behaviors is likely, at least in part, to have been a consequence of the two studies’ design, which intentionally focused on the context for disrespectful care. Neither study explicitly sought to elicit positive reports of health care and thus, both may have skewed or overstated negative reports. Moreover, strong personalities or dramatic stories can change the tenor of focus group discussions. It is possible that hearing about the negative experiences of others prompted some participants (whether health workers or community members) to overemphasize their own negative experiences. The mortality study’s reliance on snowball sampling that originated with relatives of deceased women likely resulted in some selection bias, and it is possible that people who were unhappy with maternity services volunteered as a means of voicing their dissatisfaction. The small number of medical officers as compared to nurses means that differences between the two cadres must be interpreted with caution, as they may be an artifact of the small sample size. The questions in the survey were not validated, so it is also possible that respondents interpreted the meanings of the questions somewhat differently. However, since the responses to the survey mirror the contributions of participants in the focus groups, we are confident that differences in interpretations are unlikely to be drastic enough to change the validity of the responses. Finally, qualitative reports of negative practices do not represent how often that practice actually occurs in a facility, and the survey’s focus on colleagues’ attitudes and behaviors did not allow for quantification of respondents’ own practices. On the other hand, the combined quantitative and qualitative results from both studies provide a richer picture of health worker and client perspectives. In Mannava et al.’s review of 31 studies that included health workers, about two-thirds relied solely on qualitative methods [13].

### Recommendations

Taken together, the two Namibian studies generated a number of valuable findings and impressions that can be used to identify recommendations. For starters, health workers who participated in the focus groups pinpointed three broad sets of recommendations to alleviate systemic deficiencies that contribute to disrespectful care. These included:

- Implementing measures to address heavy workloads, such as speeding up recruitment, filling vacant positions, paying more appropriate salaries, and providing stress management counselling;
- Providing structural and quality of care improvements, such as needed equipment, infrastructure upgrades, professional development, and customer care training; and
- Educating communities and the media about maternity care and health workers’ roles.

Abuya et al. [19] implemented complementary facility and community interventions in Kenya, which achieved noteworthy decreases in both subjective and objective measures of disrespect and abuse. This work suggests that multicomponent interventions are essential and should encompass policy dialogue with government, civil society, and professional associations; structural interventions to improve the working environment; training sessions with health workers focusing on values clarification and RMC; interventions to strengthen facility-community linkages and increase accountability; and mediation and resolution of reported cases of disrespect and abuse [19]. Abuya and colleagues also pointed out that meaningful and long-lasting results cannot be rushed or achieved in a short period of time [19].

Our health workers’ focus group results also suggest that interventions need to address the preservice training environment where professional socialization begins. To this end, preservice training curricula need to be reviewed. From the outset of professional education, training needs to go beyond a strictly clinical focus on knowledge-based competencies to also emphasize compassionate client-centered care [45] and professional ethics [46]. Supervisors and mentors who have emerged from the current clinically oriented training environment also need updated training. Establishing a code of conduct that is upheld by all health workers and institutional leadership is important, and accountability mechanisms to report and intervene in disrespectful care need to be developed. Finally, there is clearly room for more community education about client rights. The 1998 Patient Charter of Namibia [47], which highlights the rights of patients and the respective roles and responsibilities of patients and health workers, remains largely unknown.

## Conclusions

Societies generally attach a high value to motherhood, and the experience of health care during pregnancy and childbirth often contributes to longer-term health-care-seeking behavior and expectations for women. In recognition of the fact that women are intensely vulnerable before, during and after childbirth, it is essential that institutional childbirth be a reliably positive and safe experience. Respectful maternity care is not an optional part of skilled attendance and emergency obstetric and newborn care but a critical component that affects outcomes for mothers and their newborns. By commissioning and taking an active part in the two studies and spearheading an institutional restructuring process, Namibia’s Ministry of Health and Social Services has demonstrated its commitment to making positive changes for health workers and clients.

## Additional file

Additional file 1: Questionnaire for Health Workers. This is the questionnaire administered to health worker respondents. (DOCX 52 kb)

## Abbreviations

EN: Enrolled nurse
MO: Medical officer
MoHSS: Ministry of Health and Social Services
RMC: Respectful maternity care
RN: Registered nurse
