# Supplementary material for: Provider and client perspectives on maternity care in Namibia: results from two cross-sectional studies
Source: BMC Pregnancy Childbirth. 2018 Sep 5;18:363. doi: 10.1186/s12884-018-1999-3 (PMC6126026; doi:10.1186/s12884-018-1999-3)
Supplement: Supplementary file 1 — Questionnaire for Health Workers. This is the questionnaire administered to health worker respondents. (DOCX 52 kb) [file 12884_2018_1999_MOESM1_ESM.docx]

## Questionnaire for Health Workers

**Name of Health Facility:……………………………………………………………**

## Section 1: Background characteristics of health worker

| ***No*** | ***Questions and Filters*** | | | ***Coding Categories*** |
| --- | --- | --- | --- | --- |
|  | How old were you at your last birthday? | | | [______\|______] |
|  | What is your sex? | | | 1: Male  2: Female |
|  | Of what country are you a citizen? | | | 1: Namibian  2: Non-Namibian |
|  | If Non-Namibian: How long have you been working in Namibia as a health worker (**in years**)? | | | [______\|______] |
|  | Are you: | | | 1: Enrolled Nurse  2: Registered Nurse  3: Medical Officer  4:Other (specify): |
|  | What is your highest qualification? | | | 1: Certificate  2: Diploma  3: Degree  4: Post graduate  5: other (specify): |
|  | Where were you trained for your highest qualification? | | | 1: NHTC-WHK  2: RHTC-Onandjokwe  3: RHTC-Rundu  4: RHTC Otjiwarongo  5: UNAM-Oshakati  6: UNAM-Windhoek  7: other (specify): |
|  | In which year did you complete your highest training? | | | [_____\|_____\|_____\|_____] |
|  | Have you attended any additional training in Maternal and Neonatal health care after your pre-service training? | | | 1: Yes  2: No |
|  | What type of additional training in maternal and neonatal health care have you received post- pre-service training?  (*multiple responses allowed*) | | | 1. EmOC training: 2. Life Saving skills 3. Other (specify)… |
|  | What is your job position now? | | | 1:Maternity nurse in-charge  2: Maternity nurse  3: Matron  4: Medical Officer in Maternity  5: Principal Medical Officer  6: other (specify): |
|  | How many years of experience subsequent to completion of pre-service training do you have? | | | [______\|______] |
|  | How many years of service do you have in delivery care? | | | [______\|______] |
|  | What made you decide to become a [**choose the correct category for respondent]** registered/enrolled nurse/medical officer?  *(multiple responses allowed*) | 1: Felt a “calling” for the profession  2: Had a personal experience with the health care system that motivated me  3: A family member/influential person recommended it  4: Availability of jobs in this profession  5: Attractive salary and benefits  6: Other (specify) | | |
|  | If I had to decide over again, I would still choose to become a [registered, enrolled nurse, doctor]. | | | 1: Strongly Agree  2: Agree  3: Disagree  4: Strongly Disagree |
|  | What is your marital status? | | | 1: Married  2: Never Married  3: Divorced  4: Cohabiting  5: Widow(er) |
|  | What is your religion? | | | 1 Christian – Protestant  2. Christian – Catholic  3. Muslim  4. No specific religion  5. Other (specify): |
| **Please respond to the statements below with your level of agreement or disagreement.** | | | | |
|  | I am satisfied in a moral sense with the services we provide to patients in this maternity.  *Ref. Faye 38* | | | 1: Strongly Agree  2: Agree  3: Disagree  4: Strongly Disagree |
|  | I am satisfied that my workplace does not prevent me from practicing my religion.  *Ref. Faye 39* | | | 1: Strongly Agree  2: Agree  3: Disagree  4: Strongly Disagree |
|  | Some of the things I do at work conflict with my religious beliefs. | | | 1: Strongly Agree  2: Agree  3: Disagree  4: Strongly Disagree |
|  | **IF RESPONDENT AGREES/STRONGLY AGREES:**  Could you please tell me more about your concerns? | | | |
|  | On days when you are working in this facility, what is your residence? | | | 1: Nurse’s home  2: Own house  3: Renting  4: Sharing with friends  5: Sharing with family  6: Doctors’ flats  7: Other (specify) : |
|  | When you are working, how far away do you live from your family? | | | 1: Lives with family  2: Lives within one hour's travel from family  3: Lives less than one day but more than an hour away  4: Lives more than one day's travel away |
|  | The distance I live from my family impacts my satisfaction with my job. | | 1: Strongly Agree  2: Agree  3: Disagree  4: Strongly Disagree | |
|  | I am satisfied with living near the location where I work.  For example, are you happy to be living in this district? | | 1: Strongly Agree  2: Agree  3: Disagree  4: Strongly Disagree | |

## Section 2: Job satisfaction

**READ:** Thanks. There are many elements that combine to make a job satisfying. Now I’m going to read some statements to you. This will be a combination of questions about your job *in the maternity* and how satisfied you are in your work. After each statement, please tell me whether you agree, strongly agree, disagree, or strongly disagree. And, remember, please, that we are asking specifically about your experiences working in the maternity in this facility.

| ***No*** | ***Questions and Filters*** | ***Coding Categories*** | |
| --- | --- | --- | --- |
|  | I am satisfied with the schedule and hours that I work.  *Ref. Faye 11* | 1: Strongly Agree  2: Agree  3: Disagree  4: Strongly Disagree | |
|  | I am satisfied with the workload that I have.  *Ref. Faye 12* | 1: Strongly Agree  2: Agree  3: Disagree  4: Strongly Disagree | |
|  | I am satisfied with the balance of the workload between myself and my colleagues.  *Ref. Faye 13* | 1: Strongly Agree  2: Agree  3: Disagree  4: Strongly Disagree | |
|  | I am satisfied with the balance between the time I spend with clients and the time I spend on other activities.  *Ref. Faye 14* | 1: Strongly Agree  2: Agree  3: Disagree  4: Strongly Disagree | |
|  | I am satisfied with the support I receive from other members of the team to accomplish our tasks.  *Ref. Faye 15* | 1: Strongly Agree  2: Agree  3: Disagree  4: Strongly Disagree | |
|  | I am satisfied with the variety of tasks that I have to accomplish at work.  For example, you appreciate the diversity of the work and you not bored.  *Ref. Faye 16* | 1: Strongly Agree  2: Agree  3: Disagree  4: Strongly Disagree | |
|  | I am satisfied with the balance that exists between my tasks and my skills.  For example, you are satisfied that the tasks you are given match the skills that you have.  *Ref. Faye 17* | 1: Strongly Agree  2: Agree  3: Disagree  4: Strongly Disagree | |
|  | I am satisfied with the amount of professional responsibility that has been delegated to me.  *Ref. Faye 18* | 1: Strongly Agree  2: Agree  3: Disagree  4: Strongly Disagree | |
|  | I am satisfied with the job description for my position.  *Ref. Faye 19* | 1: Strongly Agree  2: Agree  3: Disagree  4: Strongly Disagree | |
|  | I am satisfied that my job description matches with what I actually do.  *Ref. Faye 20* | 1: Strongly Agree  2: Agree  3: Disagree  4: Strongly Disagree | |
|  | During the day shifts, there is adequate number of staff at this hospital to provide good quality maternal and neonatal health care. | 1: Strongly Agree  2: Agree  3: Disagree  4: Strongly Disagree |  |
|  | During the night shifts, there is adequate number of staff at this hospital to provide good quality maternal and neonatal health care. | 1: Strongly Agree  2: Agree  3: Disagree  4: Strongly Disagree |  |
| ***Materials, equipment and infrastructure*** | | | |
|  | We have enough blood available for transfusions when we need it.  *Ref. Faye 6* | 1: Strongly Agree  2: Agree  3: Disagree  4: Strongly Disagree | |
|  | We have enough medical equipment available to do our work. | 1: Strongly Agree  2: Agree  3: Disagree  4: Strongly Disagree | |
|  | We have enough medications available to do our work.  *Ref. Faye 7* | 1: Strongly Agree  2: Agree  3: Disagree  4: Strongly Disagree | |
|  | I am satisfied that we have enough supplies (for example, cotton wool, alcohol) available to do our work.  *Ref. Faye 8* | 1: Strongly Agree  2: Agree  3: Disagree  4: Strongly Disagree | |
|  | I am satisfied that we have enough space in the maternity ward. | 1: Strongly Agree  2: Agree  3: Disagree  4: Strongly Disagree | |
|  | I am satisfied that we have enough space in the postpartum ward. | 1: Strongly Agree  2: Agree  3: Disagree  4: Strongly Disagree | |
|  | I am satisfied that I am protected against profession-related risks (for example, exposure to HIV, physical safety).  *Ref. Faye 9* | 1: Strongly Agree  2: Agree  3: Disagree  4: Strongly Disagree | |
|  | I am satisfied that we have enough printed resources available to do our work.  *Ref. Faye 10* | 1: Strongly Agree  2: Agree  3: Disagree  4: Strongly Disagree | |
| ***Continuing education*** | | | |
|  | I am satisfied with the training that I have received and continue to receive in this position (not including pre-service training).  *Ref. Faye 26* | 1: Strongly Agree  2: Agree  3: Disagree  4: Strongly Disagree | |
|  | I am satisfied with how members of my unit are selected to participate in trainings.  *Ref. Faye 27* | 1: Strongly Agree  2: Agree  3: Disagree  4: Strongly Disagree | |
|  | I am satisfied that the trainings being offered are meeting my training needs.  *Ref. Faye 28* | 1: Strongly Agree  2: Agree  3: Disagree  4: Strongly Disagree | |
|  | I am satisfied with how I can use what I learned in trainings in my work.  *Ref. Faye 29* | 1: Strongly Agree  2: Agree  3: Disagree  4: Strongly Disagree | |
|  | I am satisfied with the level of skill that I achieve during trainings (including in-service training).  *Ref. Faye 30* | 1: Strongly Agree  2: Agree  3: Disagree  4: Strongly Disagree | |
| ***Salary and benefits*** | | | |
|  | I am satisfied with the amount of my salary.  *Ref. Faye 1* | 1: Strongly Agree  2: Agree  3: Disagree  4: Strongly Disagree |  |
|  | I am satisfied with the other bonuses and benefits that I receive.  *Ref. Faye 2* | 1: Strongly Agree  2: Agree  3: Disagree  4: Strongly Disagree |  |
|  | I am satisfied that what I receive in salary and benefits provides me what I need.  *Ref. Faye 3* | 1: Strongly Agree  2: Agree  3: Disagree  4: Strongly Disagree |  |
|  | I am satisfied that the amount of my salary is commensurate with my skills.  *Ref. Faye 4* | 1: Strongly Agree  2: Agree  3: Disagree  4: Strongly Disagree |  |
|  | I am satisfied with the amount of my salary as compared to my workload.  *Ref. Faye 5* | 1: Strongly Agree  2: Agree  3: Disagree  4: Strongly Disagree |  |
|  | I am satisfied with how regularly I receive my salary.  *Ref. Faye 41* | 1: Strongly Agree  2: Agree  3: Disagree  4: Strongly Disagree |  |
|  | I am satisfied with the stability of my employment (certainty/uncertainty that I will retain/lose my job).  *Ref. Faye 42* | 1: Strongly Agree  2: Agree  3: Disagree  4: Strongly Disagree |  |
|  | I am satisfied with how regularly I receive my bonuses.  *Ref. Faye 43* | 1: Strongly Agree  2: Agree  3: Disagree  4: Strongly Disagree |  |
|  | Lack of employer-provided transport negatively affects my feelings of satisfaction about my job. | 1: Strongly Agree  2: Agree  3: Disagree  4: Strongly Disagree |  |
|  | When public transport is delayed it negatively affects my work performance. | 1: Strongly Agree  2: Agree  3: Disagree  4: Strongly Disagree |  |
|  | Lack of employer-provided accommodation negatively affects my feelings of satisfaction about my job. | 1: Strongly Agree  2: Agree  3: Disagree  4: Strongly Disagree |  |
|  | Have you ever worked in the private sector? | 1: Yes  2: No |  |
|  | If you were offered a job today in the private sector with the same salary you receive now in the public sector, would you accept it? | 1: Yes  2: No |  |
|  | **For both yes and no responses:** Why? | |  |
| ***Working environment and team factors*** | | | |
|  | Working with less experienced colleagues negatively affects my feelings of satisfaction about my job. | 1: Strongly Agree  2: Agree  3: Disagree  4: Strongly Disagree |  |
|  | Working with more experienced colleagues negatively affects my feelings of satisfaction about my job. | 1: Strongly Agree  2: Agree  3: Disagree  4: Strongly Disagree |  |
|  | I am satisfied with the way that the members of my unit get along with each other.  *Ref. Faye 21* | 1: Strongly Agree  2: Agree  3: Disagree  4: Strongly Disagree |  |
|  | I feel most of my work colleagues are approachable and openly discuss work-related problems. | 1: Strongly Agree  2: Agree  3: Disagree  4: Strongly Disagree |  |
|  | Sometimes I have trouble communicating with colleagues who are of a different cadre than mine.  For example, a nurse talking to a doctor or vice versa. | 1: Strongly Agree  2: Agree  3: Disagree  4: Strongly Disagree |  |
|  | I feel most of my colleagues are respectful of patients when providing maternal and neonatal health care. | 1: Strongly Agree  2: Agree  3: Disagree  4: Strongly Disagree |  |
|  | I have seen colleagues or know of incidences where patients were mistreated at my institution. | 1: Strongly Agree  2: Agree  3: Disagree  4: Strongly Disagree |  |
|  | Co-workers’ attitudes towards each other negatively affect my feelings of satisfaction about my job. | 1: Strongly Agree  2: Agree  3: Disagree  4: Strongly Disagree |  |
| ***Management and supervision*** | | | |
|  | In general, I am satisfied with how positive feedback is provided in my unit.  *Ref. Faye 32* | 1: Strongly Agree  2: Agree  3: Disagree  4: Strongly Disagree |  |
|  | I feel that my current manager is supportive and approachable. | 1: Strongly Agree  2: Agree  3: Disagree  4: Strongly Disagree |  |
|  | I feel that my current manager is adequately present. | 1: Strongly Agree  2: Agree  3: Disagree  4: Strongly Disagree |  |
|  | I am satisfied with the level of recognition of the quality of my work that I receive from my colleagues. I am satisfied with the recognition I get from my colleagues about the quality of my work.  *Ref. Faye 22* | 1: Strongly Agree  2: Agree  3: Disagree  4: Strongly Disagree |  |
|  | I am satisfied with the level of recognition of the quality of my work that I receive from my superiors.  *Ref. Faye 23* | 1: Strongly Agree  2: Agree  3: Disagree  4: Strongly Disagree |  |
|  | I am satisfied with the way employees are assessed before being considered for a promotion.  *Ref. Faye 24* | 1: Strongly Agree  2: Agree  3: Disagree  4: Strongly Disagree |  |
|  | I am satisfied with the respect paid to me by my superiors.  *Ref. Faye 25* | 1: Strongly Agree  2: Agree  3: Disagree  4: Strongly Disagree |  |
|  | I am satisfied with the opportunities provided to participate in problem-solving to improve our work.  *Ref. Faye 32* | 1: Strongly Agree  2: Agree  3: Disagree  4: Strongly Disagree |  |
|  | I am satisfied with the information I receive regarding what is going on within our unit/service.  *Ref. Faye 33* | 1: Strongly Agree  2: Agree  3: Disagree  4: Strongly Disagree |  |
|  | I am satisfied with the information I receive regarding things happening in the facility (problems, activities, decisions being made, financial management).  *Ref. Faye 34* | 1: Strongly Agree  2: Agree  3: Disagree  4: Strongly Disagree |  |
|  | I am satisfied with the transparency with which the financial resources are managed in this facility.  *Ref. Faye 35* | 1: Strongly Agree  2: Agree  3: Disagree  4: Strongly Disagree |  |
|  | I am satisfied with the quality of the work that I do.  *Ref. Faye 37* | 1: Strongly Agree  2: Agree  3: Disagree  4: Strongly Disagree |  |
|  | I am satisfied with the outcomes of child deliveries in my unit (health of mothers and newborns).  *Ref. Faye 36* | 1: Strongly Agree  2: Agree  3: Disagree  4: Strongly Disagree |  |

## Section 3: Experiences providing care in the maternity – obstacles and facilitators

**READ:** Next is a series of statements about situations that you may possibly have experienced or seen during the process of providing care to pregnant women and infants in the maternity service. After each statement, please tell me whether you agree, strongly agree, disagree, or strongly disagree. There is no right or wrong answer to these questions. We are looking to understand your real world experiences. Your responses will not be shared with your colleagues or your supervisors in a way in which you can be identified.

| ***No*** | ***Questions*** | ***Coding categories*** |
| --- | --- | --- |
| ***Community and social support*** | | |
| 3.1 | I feel that this community appreciates my work and service in this facility. | 1: Strongly Agree  2: Agree  3: Disagree  4: Strongly Disagree |
| 3.2 | I am satisfied with the image that my profession has in this community.  *Ref. Faye 40* | 1: Strongly Agree  2: Agree  3: Disagree  4: Strongly Disagree |
| 3.3 | I have adequate support from family and/or friends to discuss work-related problems. | 1: Strongly Agree  2: Agree  3: Disagree  4: Strongly Disagree |
| 3.4 | I speak the local language of the community within which I work. | 1: Strongly Agree  2: Agree  3: Disagree  4: Strongly Disagree |
| 3.5 | I have friends in the community that is served by this facility. | 1: Strongly Agree  2: Agree  3: Disagree  4: Strongly Disagree |
| 3.6 | I was welcomed by the village leaders of the community that is served by this facility when I started. | 1: Strongly Agree  2: Agree  3: Disagree  4: Strongly Disagree |
| 3.7 | I feel that I understand well the community that is served by this facility. | 1: Strongly Agree  2: Agree  3: Disagree  4: Strongly Disagree |
| 3.8 | I am less satisfied with my job now than I was when I started working after pre-service training. | 1: Strongly Agree  2: Agree  3: Disagree  4: Strongly Disagree |
| 3.9 | It is possible that a woman will be sent home too early after delivery because there are not enough beds. | 1: Strongly Agree  2: Agree  3: Disagree  4: Strongly Disagree |
| 3.10 | If a woman comes to the facility in early labor, she can be sent home because there is no room for her in the facility. | 1: Strongly Agree  2: Agree  3: Disagree  4: Strongly Disagree |
| 3.11 | It is not worth it to explain procedures to uneducated patients because they will not be able to understand. | 1: Strongly Agree  2: Agree  3: Disagree  4: Strongly Disagree |
| 3.12 | Sometimes you have to yell at a woman in labor because she is not pushing hard enough. | 1: Strongly Agree  2: Agree  3: Disagree  4: Strongly Disagree |
| 3.13 | Pregnant women less than 20 years old are sometimes treated negatively compared to the treatment of older women in my health facility. | 1: Strongly Agree  2: Agree  3: Disagree  4: Strongly Disagree |
| 3.14 | Women should be allowed to have friends and family with them in the delivery room to help them through labor and birth. | 1: Strongly Agree  2: Agree  3: Disagree  4: Strongly Disagree |
| 3.15 | Sometimes I get upset at a woman when I see her becoming pregnant over and over again. | 1: Strongly Agree  2: Agree  3: Disagree  4: Strongly Disagree |
| 3.16 | Patients should have a way to give feedback to the facility about the quality of care they receive. | 1: Strongly Agree  2: Agree  3: Disagree  4: Strongly Disagree |
| 3.17 | Sometimes I have served so many pregnant women that I am too tired to give the next woman good service. | 1: Strongly Agree  2: Agree  3: Disagree  4: Strongly Disagree |
| 3.18 | Sometimes pinching or slapping a woman in labor can succeed in getting her to push harder. | 1: Strongly Agree  2: Agree  3: Disagree  4: Strongly Disagree |
| 3.19 | Sometimes, if you push hard on the mother’s abdomen, you can make the baby come faster. | 1: Strongly Agree  2: Agree  3: Disagree  4: Strongly Disagree |
| 3.20 | Patients complain too much; they don’t understand how hard we are working. | 1: Strongly Agree  2: Agree  3: Disagree  4: Strongly Disagree |
| 3.21 | If I see another provider disrespecting a patient, I feel I should stop him/her. | 1: Strongly Agree  2: Agree  3: Disagree  4: Strongly Disagree |
| 3.22 | It is not always necessary to use anesthetics when repairing an episiotomy cut or vaginal tear. | 1: Strongly Agree  2: Agree  3: Disagree  4: Strongly Disagree |
| 3.23 | Women in labor should not be allowed to cry and yell about the pain. | 1: Strongly Agree  2: Agree  3: Disagree  4: Strongly Disagree |
| 3.24 | A provider does not need to stay with a woman in late labor for every minute of the time. | 1: Strongly Agree  2: Agree  3: Disagree  4: Strongly Disagree |
| 3.25 | I will not deliver the baby of an HIV+ woman. | 1: Strongly Agree  2: Agree  3: Disagree  4: Strongly Disagree |
| 3.26 | Sometimes if you threaten a woman in labor with having a caesarian section, she will succeed better in delivering normally. | 1: Strongly Agree  2: Agree  3: Disagree  4: Strongly Disagree |
| 3.27 | A woman who has given birth in our facility must pay her bill before we will allow her and her baby to leave the facility, even if it takes days. | 1: Strongly Agree  2: Agree  3: Disagree  4: Strongly Disagree |
| 3.28 | Communities should have a way to give feedback to the facility about the quality of care they give. | 1: Strongly Agree  2: Agree  3: Disagree  4: Strongly Disagree |
| 3.29 | It is easier assisting educated women when they come for maternal and neonatal health care than women who are not educated. | 1: Strongly Agree  2: Agree  3: Disagree  4: Strongly Disagree |
| 3.30 | Sometimes you just have to do a procedure, like an episiotomy, even if the patient doesn’t understand what it is. | 1: Strongly Agree  2: Agree  3: Disagree  4: Strongly Disagree |
| 3.31 | Sometimes I get angry at a woman when she knows she is HIV-positive and yet has become pregnant. | 1: Strongly Agree  2: Agree  3: Disagree  4: Strongly Disagree |
| 3.32 | If an unmarried woman comes in to the facility pregnant, it is a provider’s responsibility to counsel her that she has been behaving badly. | 1: Strongly Agree  2: Agree  3: Disagree  4: Strongly Disagree |
| 3.33 | Newborns can be cared for by anyone in the first few hours; they don’t need to be by their mother. | 1: Strongly Agree  2: Agree  3: Disagree  4: Strongly Disagree |
| 3.34 | Women who don’t want to breastfeed should not be encouraged; it's a personal choice. | 1: Strongly Agree  2: Agree  3: Disagree  4: Strongly Disagree |
| 3.35 | I always tell the patient my findings during an examination. | 1: Strongly Agree  2: Agree  3: Disagree  4: Strongly Disagree |
| 3.36 | I have seen colleagues handle newborns roughly or without care when their mothers are not in the room. | 1: Strongly Agree  2: Agree  3: Disagree  4: Strongly Disagree |
| 3.37 | Sometimes it is better if a newborn that is ill or has a deformity is allowed to die. | 1: Strongly Agree  2: Agree  3: Disagree  4: Strongly Disagree |
| 3.38 | Sometimes patients from certain ethnic groups are treated differently than others in my health facility. | 1: Strongly Agree  2: Agree  3: Disagree  4: Strongly Disagree |
| 3.39 | I always explain what I am doing before touching a client, especially for procedures such as a vaginal or breast exam, injection, or abdominal exam. | 1: Strongly Agree  2: Agree  3: Disagree  4: Strongly Disagree |
| 3.40 | I tell other providers when an HIV+ mother is delivering or seeking services because they have a right to know. | 1: Strongly Agree  2: Agree  3: Disagree  4: Strongly Disagree |
| 3.41 | I tell other patients when an HIV+ mother is delivering or seeking services because they have a right to know. | 1: Strongly Agree  2: Agree  3: Disagree  4: Strongly Disagree |
| 3.42 | It is hard to remain positive about providing care in the maternity service when you are woken up in the night. | 1: Strongly Agree  2: Agree  3: Disagree  4: Strongly Disagree |
| 3.43 | I do not discuss personal details about the patient in public. | 1: Strongly Agree  2: Agree  3: Disagree  4: Strongly Disagree |
| 3.44 | I require laboring women to deliver on their backs (lithotomy position). | 1: Strongly Agree  2: Agree  3: Disagree  4: Strongly Disagree |
| 3.45 | During examinations I draw curtains between beds, if possible. | 1: Strongly Agree  2: Agree  3: Disagree  4: Strongly Disagree |
| 3.46 | When a woman speaks my language it is easier for me to assist her in delivering maternal and neonatal health care. (during labor and delivery?) | 1: Strongly Agree  2: Agree  3: Disagree  4: Strongly Disagree |
| 3.47 | It is easier for me to assist a woman from my ethnic group. | 1: Strongly Agree  2: Agree  3: Disagree  4: Strongly Disagree |
| 3.48 | It is easier for me to assist a woman who belongs to my religion. | 1: Strongly Agree  2: Agree  3: Disagree  4: Strongly Disagree |
| 3.49 | Some providers at this facility treat women of low social status more poorly than other women of higher status. | 1: Strongly Agree  2: Agree  3: Disagree  4: Strongly Disagree |
| 3.50 | A patient’s age does not affect the way I provide maternal and neonatal health care. | 1: Strongly Agree  2: Agree  3: Disagree  4: Strongly Disagree |
| 3.51 | A patient’s education level does not affect the way I provide maternal and neonatal health care. | 1: Strongly Agree  2: Agree  3: Disagree  4: Strongly Disagree |
| 3.52 | In my family it is a taboo to work in maternity and neonatal care. | 1: Strongly Agree  2: Agree  3: Disagree  4: Strongly Disagree |
| 3.53 | We need to make improvements in delivering maternal and neonatal duties in order to meet patients’ needs. | 1: Strongly Agree  2: Agree  3: Disagree  4: Strongly Disagree |
| 3.54 | What improvements do you think are needed? (*applicable to those who Agree or Strongly agree* *in 3.45*) | |

## Section 4: Regulatory frameworks

**READ:** I really appreciate your patience. This is our final section. We have a few questions about some policies and regulations that exist in Namibia.

| ***No*** | ***Questions*** | ***Coding categories*** | |
| --- | --- | --- | --- |
| 4.1 | I am familiar with the content of the following regulatory frameworks:   - Nursing Act of Namibia (Act 8 of 2004) - Patients Charter of Namibia - Medical and Dental Act - Health Professional Council of Namibia - Policy guidelines on Obstetric Care | Yes | No |
|  |  | 1 | 0 |
|  |  | 1 | 0 |
|  |  | 1 | 0 |
|  |  | 1 | 0 |
|  |  | 1 | 0 |
| 4.2 | The Nursing Act of Namibia positively affects the way I provide maternal and neonatal health care. | 1: Strongly Agree  2: Agree  3: Disagree  4: Strongly Disagree | |
| 4.3 | The Patients Charter of Namibia positively affects the way I provide maternal and neonatal health care. | 1: Strongly Agree  2: Agree  3: Disagree  4: Strongly Disagree | |
| 4.4 | The Medical and Dental Act positively affects the way I provide maternal and neonatal health care. | 1: Strongly Agree  2: Agree  3: Disagree  4: Strongly Disagree | |
| 4.5 | The Health Professional Council of Namibia Act positively affects the way I provide maternal and neonatal health care. | 1: Strongly Agree  2: Agree  3: Disagree  4: Strongly Disagree | |
| 4.6 | The policy guidelines on Obstetric Care positively affect the way I provide maternal and neonatal health care. | 1: Strongly Agree  2: Agree  3: Disagree  4: Strongly Disagree | |
| 4.7 | The regulatory frameworks positively affect the way I deliver maternal and neonatal health care. | 1: Strongly Agree  2: Agree  3: Disagree  4: Strongly Disagree | |
| 4.8 | I find the Nursing Act restrictive towards delivery of maternal and neonatal health care. | 1: Strongly Agree  2: Agree  3: Disagree  4: Strongly Disagree | |
| 4.9 | I find the Medical and Dental Act restrictive towards delivery of maternal and neonatal health care. | 1: Strongly Agree  2: Agree  3: Disagree  4: Strongly Disagree | |
| 4.10 | I find the Health Professional Council Act restrictive towards delivery of maternal and neonatal health care. | 1: Strongly Agree  2: Agree  3: Disagree  4: Strongly Disagree | |
| 4.11 | I find the Patients’ Charter restrictive towards delivery of maternal and neonatal health care. | 1: Strongly Agree  2: Agree  3: Disagree  4: Strongly Disagree | |
| 4.12 | I find the Policy Guidelines on Obstetrics Care restrictive towards the delivery of maternal and neonatal care. | 1: Strongly Agree  2: Agree  3: Disagree  4: Strongly Disagree | |
| 4.13 | I consider the rights of a patient as stated in the Patient Charter of Namibia when delivering maternal and neonatal care. | 1: Strongly Agree  2: Agree  3: Disagree  4: Strongly Disagree | |

**Section 5: Knowledge of lifesaving skills**

We would like to determine what areas of knowledge need reinforcement. Next are a series of statements about providing lifesaving skills during childbirth. Please answer true or false to each statement.

| ***No*** | ***Questions*** | ***Coding categories*** |
| --- | --- | --- |
| ***Community and social support*** | | |
| 5.1 | During active labor when the woman has 3 – 4 contractions in 10 minutes, she should use slow, deep breathing to help her relax. | 1: True  2: False |
| 5.2 | During the active phase of labor, the first vaginal exam should be plotted on the alert line of the partograph. | 1: True  2: False |
| 5.3 | ­­­Women should be instructed to push forcefully with each contraction as soon as the cervix is fully dilated. | 1: True  2: False |
| 5.4 | Active management of third stage of labor should be performed only on woman who are high risk for postpartum hemorrhage. | 1: True  2: False |
| 5.5 | The time between the alert line and the action line is 3 hours. | 1: True  2: False |
| 5.6 | Immediate postpartum hemorrhage is most commonly due to lacerations. | 1: True  2: False |
| 5.7 | Starting an IV line is the **first** step in management of atonic uterus. | 1: True  2: False |
| 5.8 | A newborn baby with asphyxia can be resusitated effectively without oxygen. | 1: True  2: False |
| 5.9 | According to evidence, the umbilical stump should be cleaned daily with alcohol. | 1: True  2: False |
| 5.10 | A baby can be warmed more quickly by skin-to-skin care with the mother instead of in an incubator. | 1: True  2: False |
| 5.11 | 10 IU oxytocin should be provided **after** clamping and cutting the cord. | 1: True  2: False |
| 5.12 | Standard precautions are used for the care of all persons, clients and staff, if they are infected or not. | 1: True  2: False |
| 5.13 | Decontamination of instruments with 0.5% chlorine solution for 10 minutes before cleaning kills most microorganisms including Hepatitis B, Hepatitis C, and HIV viruses. | 1: True  2: False |
| 5.14 | Before placing a disposable needle and syringe in a puncture proof container or box, you should first carefully re-cap the needle. | 1: True  2: False |
| 5.15 | After delivery of the placenta it is important to inspect the placenta before massaging the uterus. | 1: True  2: False |
| 5.16 | You should encourage a woman in labor to drink at least every 2 hours. | 1: True  2: False |
| 5.17 | A woman is in active labor. Her membranes rupture spontaneously and you see heavy meconium stained amniotic fluid. You should check the fetal heart every 30 minutes. | 1: True  2: False |
| 5.18 | A primigravida should dilate at least 1 cm per hour in active labor. | 1: True  2: False |
| 5.19 | Delivering a woman in the left lateral position increases the chance her perineum will tear. | 1: True  2: False |
| 5.20 | If a baby needs newborn resuscitation, ventilation should be started with 1 minute of birth. | 1: True  2: False |

**Thank you for your time and patience in completing this survey. Your responses will help the MOHSS to understand better the realities of providing maternal and neonatal care in Namibia.**
